# (2,2′-Bipyridine)(pyridine-2,6-dicarboxyl­ato)oxidovanadium(IV) ethanol monosolvate

**DOI:** 10.1107/S1600536811002376

**Published:** 2011-01-22

**Authors:** Hossein Aghabozorg, Elnaz Tavakoli, Masoud Mirzaei

**Affiliations:** a Faculty of Chemistry, Islamic Azad University, North Tehran Branch, Tehran, Iran; b Department of Chemistry, School of Sciences, Ferdowsi University of Mashhad, Mashhad 917791436, Iran

## Abstract

In the title compound, [V(C_7_H_3_NO_4_)O(C_10_H_8_N_2_)]·C_2_H_5_OH, the V^IV^ atom exhibits a distorted octa­hedral coordination environment formed by two pyridyl N atoms of 2,2′-bipyridine (bpy), the vanadyl O atom, and two carboxyl­ate O atoms and one pyridyl N atom of the tridentate pyridine-2,6-dicarboxyl­ate (pydc^2−^) ligand. The pyridyl N atom of the pydc^2−^ anion and one pyridyl N atom of bpy occupy the axial positions. O—H⋯O hydrogen bonds involving the ethanol solvent mol­ecule as donor and a carboxyl­ate O atom as acceptor atoms, as well as C—H⋯O hydrogen bonds, together with π–π stacking inter­actions between adjacent aromatic rings (average centroid–centroid distance = 3.577 Å), seem to be effective in the stabilization of the crystal packing, resulting in the formation of a three-dimensional structure.

## Related literature

For general background to proton-transfer compounds and their complexes, see: Aghabozorg *et al.* (2008 ▶). For related structures with V^IV^, see: Therrien *et al.* (2002 ▶); Okabe & Muranishi (2002 ▶). 
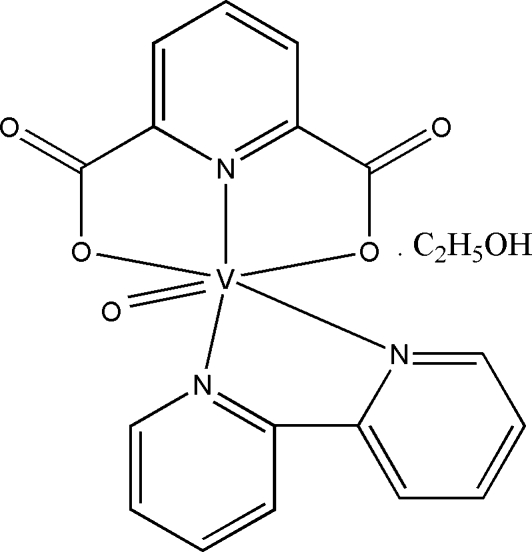


## Experimental

### 

#### Crystal data

[V(C_7_H_3_NO_4_)O(C_10_H_8_N_2_)]·C_2_H_6_O
*M* *_r_* = 434.30
Monoclinic, 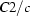

*a* = 23.246 (2) Å
*b* = 11.2179 (10) Å
*c* = 13.9440 (16) Å
β = 97.247 (9)°
*V* = 3607.1 (6) Å^3^
*Z* = 8
Mo *K*α radiation
μ = 0.60 mm^−1^
*T* = 296 K
0.27 × 0.23 × 0.02 mm

#### Data collection

Stoe IPDS II Image Plate diffractometer
Absorption correction: multi-scan (*MULABS* in *PLATON*; Spek, 2009 ▶) *T*_min_ = 0.864, *T*_max_ = 1.000
7321 measured reflections
2959 independent reflections
2124 reflections with *I* > 2*I*)
*R*_int_ = 0.063

#### Refinement

*R*[*F* ^2^ > 2σ(*F* ^2^)] = 0.058
*wR*(*F* ^2^) = 0.144
*S* = 1.03
2959 reflections
263 parameters
18 restraints
H-atom parameters constrained
Δρ_max_ = 0.40 e Å^−3^
Δρ_min_ = −0.47 e Å^−3^

### 

Data collection: *X-AREA* (Stoe & Cie, 2005 ▶); cell refinement: *X-AREA*; data reduction: *X-AREA*; program(s) used to solve structure: *SHELXTL* (Sheldrick, 2008 ▶); program(s) used to refine structure: *SHELXTL*; molecular graphics: *SHELXTL*; software used to prepare material for publication: *SHELXTL* and *PLATON* (Spek, 2009 ▶).

## Supplementary Material

Crystal structure: contains datablocks global, I. DOI: 10.1107/S1600536811002376/wm2448sup1.cif

Structure factors: contains datablocks I. DOI: 10.1107/S1600536811002376/wm2448Isup2.hkl

Additional supplementary materials:  crystallographic information; 3D view; checkCIF report

## Figures and Tables

**Table 1:** Selected bond lengths (Å)

V1—O5	1.586 (3)
V1—N3	2.020 (3)
V1—O1	2.021 (3)
V1—O2	2.035 (3)
V1—N1	2.130 (3)
V1—N2	2.304 (3)

**Table 2:** Hydrogen-bond geometry (Å, °)

*D*—H⋯*A*	*D*—H	H⋯*A*	*D*⋯*A*	*D*—H⋯*A*
O6—H6⋯O4^i^	0.82	2.00	2.815 (6)	173
C1—H1⋯O6^ii^	0.93	2.50	3.216 (7)	134
C4—H4⋯O1^iii^	0.93	2.47	3.207 (6)	137
C9—H9⋯O6^iv^	0.93	2.50	3.220 (9)	135
C12—H12⋯O5^v^	0.93	2.46	3.374 (5)	166
C14—H14⋯O5^vi^	0.93	2.52	3.146 (6)	125

Symmetry codes: (i) 
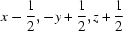
; (ii) 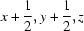; (iii) 
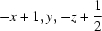
; (iv) 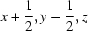; (v) 
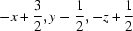
; (vi) 
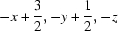
.

**Fig. 1.**
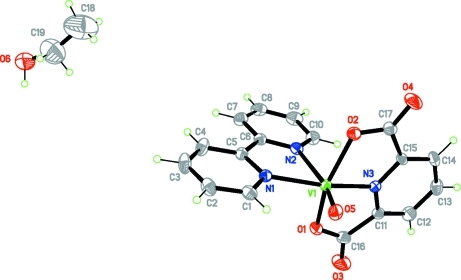
The molecular structure of the title compound with displacement ellipsoids drawn at the 50% probability level.

**Fig. 2.**
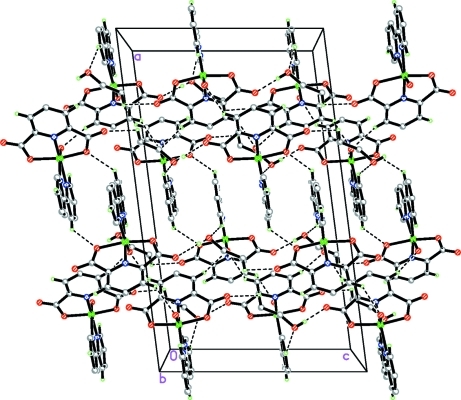
The packing diagram of the title compound. The intermolecular classical O—H···O and non-classical C—H···O hydrogen bonds are shown as dashed lines.

## supplementary crystallographic information

### Comment

2,2'-Bipyridine (bipy) is a bidentate chelating ligand, forming complexes with many transition metals. Ruthenium and platinum complexes of bipy exhibit intense luminescence, which may have practical applications. In continuation of previous works containing V^IV^complexes and various basic ligands (Aghabozorg *et al.*, 2008; Therrien *et al.*, 2002; Okabe & Muranishi, 2002), we report preparation and the crystal structure of the title compound, [VO(C_10_H_8_N_2_)(C_7_H_3_NO_4_)]^.^C_2_H_5_OH, (I).

In the structure of compound (I) the V^IV^ atom has a distorted octahedral coordination environment formed by two pyridyl N atoms of 2,2'-bipyridine (C_10_H_8_N_2_ or bpy), one O atom of the vanadyl group, and two carboxylate O atoms and one pyridyl N atom of the tridentate pyridine-2,6-dicarboxylate (C_7_H_3_NO_4_ or pydc^2-^) ligand. The pyridyl N3 atom of pydc^2-^ and the pyridyl N1 atom of bpy occupy the axial position (Fig. 1). In the crystalline network of (I), O—H···O hydrogen bonds involving the ethanol solvent and C—H···O hydrogen bonds, together with π—π stacking interactions between adjacent aromatic rings [average centroid-to-centroid distance 3.577 Å], seem to be effective in the stabilization of the crystal packing, resulting in the formation of a three-dimensional structure. In this network, layers of pydc^2-^ and bpy are alternatingly repeated in the *bc* plane (Fig. 2).

### Experimental

The reaction of vanadium^III^ chloride (78 mg, 0.5 mmol), bpy (156 mg, 1 mmol) and pydcH_2_ (167 mg, 1 mmol) in a 1:2:2 molar ratio in ethanolic/aqueous solution resulted in the formation of green platy [VO(bpy)(pydc)]^.^C_2_H_5_OH crystals.

### Refinement

All H atoms were positioned geometrically with C—H = 0.93–0.97Å and constrained to refine with the parent atoms with *U*_iso_(H) = 1.2 (aromatic and methylene H atoms) or 1.5 (methyl H atoms) *U*_eq_(C), except for the H atom of the ethanol hydroxy group which was positioned under consideration of a rotating O—H group with *U*_iso_(H) = 1.5 *U*_eq_(O) and O—H = 0.82 Å.

### Crystal data

**Table:**

[V(C_7_H_3_NO_4_)O(C_10_H_8_N_2_)]·C_2_H_6_O	*F*(000) = 1784
*M**_r_* = 434.30	*D*_x_ = 1.599 Mg m^−^^3^
Monoclinic, *C*2/*c*	Mo *K*α radiation, λ = 0.71073 Å
Hall symbol: -C 2yc	Cell parameters from 2000 reflections
*a* = 23.246 (2) Å	θ = 1.7–29.6°
*b* = 11.2179 (10) Å	µ = 0.60 mm^−^^1^
*c* = 13.9440 (16) Å	*T* = 296 K
β = 97.247 (9)°	Plate, green
*V* = 3607.1 (6) Å^3^	0.27 × 0.23 × 0.02 mm
*Z* = 8	

### Data collection

**Table:**

Stoe IPDS II Image Plate diffractometer	2959 independent reflections
Radiation source: fine-focus sealed tube	2124 reflections with *I* > 2˘*I*)
graphite	*R*_int_ = 0.063
Detector resolution: 0.15 mm pixels mm^-1^	θ_max_ = 25.0°, θ_min_ = 1.8°
ω scans	*h* = −27→27
Absorption correction: multi-scan (*MULABS* in *PLATON*; Spek, 2009)	*k* = −13→11
*T*_min_ = 0.864, *T*_max_ = 1.000	*l* = −16→16
7321 measured reflections	

### Refinement

**Table:**

Refinement on *F*^2^	Primary atom site location: structure-invariant direct methods
Least-squares matrix: full	Secondary atom site location: difference Fourier map
*R*[*F*^2^ > 2σ(*F*^2^)] = 0.058	Hydrogen site location: inferred from neighbouring sites
*wR*(*F*^2^) = 0.144	H-atom parameters constrained
*S* = 1.03	*w* = 1/[σ^2^(*F*_o_^2^) + (0.0798*P*)^2^] where *P* = (*F*_o_^2^ + 2*F*_c_^2^)/3
2959 reflections	(Δ/σ)_max_ = 0.001
263 parameters	Δρ_max_ = 0.40 e Å^−^^3^
18 restraints	Δρ_min_ = −0.47 e Å^−^^3^

### Special details

**Table:**

Geometry. All e.s.d.'s (except the e.s.d. in the dihedral angle between two l.s. planes) are estimated using the full covariance matrix. The cell e.s.d.'s are taken into account individually in the estimation of e.s.d.'s in distances, angles and torsion angles; correlations between e.s.d.'s in cell parameters are only used when they are defined by crystal symmetry. An approximate (isotropic) treatment of cell e.s.d.'s is used for estimating e.s.d.'s involving l.s. planes.
Refinement. Refinement of *F*^2^ against ALL reflections. The weighted *R*-factor *wR* and goodness of fit *S* are based on *F*^2^, conventional *R*-factors *R* are based on *F*, with *F* set to zero for negative *F*^2^. The threshold expression of *F*^2^ > σ(*F*^2^) is used only for calculating *R*-factors(gt) *etc*. and is not relevant to the choice of reflections for refinement. *R*-factors based on *F*^2^ are statistically about twice as large as those based on *F*, and *R*- factors based on ALL data will be even larger.

### Fractional atomic coordinates and isotropic or equivalent isotropic displacement parameters (Å^2^)

**Table:**

	*x*	*y*	*z*	*U*_iso_*/*U*_eq_	
V1	0.62729 (3)	0.30154 (6)	0.11171 (5)	0.0319 (2)	
O1	0.64617 (13)	0.2637 (3)	0.2539 (2)	0.0460 (8)	
O2	0.62335 (12)	0.2534 (3)	−0.0297 (2)	0.0407 (7)	
O3	0.70520 (19)	0.1657 (4)	0.3648 (2)	0.0789 (13)	
O4	0.67163 (17)	0.1626 (4)	−0.1379 (2)	0.0663 (11)	
O5	0.66057 (13)	0.4245 (3)	0.1080 (2)	0.0495 (9)	
N1	0.54682 (14)	0.3905 (3)	0.1173 (2)	0.0336 (8)	
N2	0.55688 (14)	0.1580 (3)	0.1169 (3)	0.0365 (9)	
N3	0.68851 (13)	0.1735 (3)	0.1134 (2)	0.0324 (8)	
C1	0.5446 (2)	0.5111 (4)	0.1194 (3)	0.0441 (11)	
H1	0.5787	0.5543	0.1183	0.053*	
C2	0.4937 (2)	0.5712 (5)	0.1233 (4)	0.0531 (13)	
H2	0.4937	0.6540	0.1251	0.064*	
C3	0.4433 (2)	0.5103 (5)	0.1243 (4)	0.0588 (14)	
H3	0.4085	0.5507	0.1264	0.071*	
C4	0.44461 (19)	0.3875 (5)	0.1222 (4)	0.0518 (13)	
H4	0.4106	0.3440	0.1228	0.062*	
C5	0.49684 (16)	0.3294 (4)	0.1192 (3)	0.0353 (10)	
C6	0.50287 (17)	0.1984 (4)	0.1193 (3)	0.0356 (10)	
C7	0.45626 (19)	0.1219 (4)	0.1245 (3)	0.0479 (12)	
H7	0.4191	0.1520	0.1257	0.057*	
C8	0.4662 (2)	0.0011 (5)	0.1280 (4)	0.0551 (14)	
H8	0.4356	−0.0515	0.1317	0.066*	
C9	0.5213 (2)	−0.0420 (5)	0.1261 (4)	0.0559 (14)	
H9	0.5286	−0.1235	0.1283	0.067*	
C10	0.5657 (2)	0.0393 (4)	0.1206 (4)	0.0474 (12)	
H10	0.6031	0.0106	0.1195	0.057*	
C11	0.71366 (17)	0.1325 (4)	0.1987 (3)	0.0374 (10)	
C12	0.7551 (2)	0.0444 (5)	0.2017 (4)	0.0528 (13)	
H12	0.7731	0.0158	0.2606	0.063*	
C13	0.7693 (2)	−0.0006 (5)	0.1154 (4)	0.0547 (13)	
H13	0.7973	−0.0600	0.1161	0.066*	
C14	0.74242 (18)	0.0421 (4)	0.0280 (4)	0.0456 (11)	
H14	0.7519	0.0122	−0.0302	0.055*	
C15	0.70108 (16)	0.1302 (4)	0.0292 (3)	0.0331 (10)	
C16	0.6882 (2)	0.1901 (5)	0.2804 (3)	0.0477 (12)	
C17	0.66328 (19)	0.1852 (4)	−0.0549 (3)	0.0418 (11)	
C18	0.1764 (7)	0.2303 (15)	0.0306 (11)	0.223 (7)	
H18D	0.2105	0.2775	0.0274	0.334*	
H18B	0.1481	0.2487	−0.0236	0.334*	
H18C	0.1863	0.1473	0.0290	0.334*	
C19	0.1528 (6)	0.2564 (11)	0.1197 (11)	0.165 (5)	
H19C	0.1874	0.2380	0.1635	0.199*	
H19B	0.1537	0.3426	0.1145	0.199*	
O6	0.1146 (3)	0.2505 (6)	0.1859 (5)	0.117 (2)	
H6	0.1310	0.2704	0.2390	0.175*	

### Atomic displacement parameters (Å^2^)

**Table:**

	*U*^11^	*U*^22^	*U*^33^	*U*^12^	*U*^13^	*U*^23^
V1	0.0293 (3)	0.0323 (4)	0.0341 (4)	−0.0005 (3)	0.0042 (3)	−0.0012 (3)
O1	0.0457 (16)	0.058 (2)	0.0345 (17)	0.0043 (15)	0.0067 (14)	−0.0067 (15)
O2	0.0434 (16)	0.0462 (18)	0.0318 (16)	0.0035 (14)	0.0016 (13)	0.0045 (14)
O3	0.103 (3)	0.100 (3)	0.0302 (18)	0.021 (3)	−0.005 (2)	0.004 (2)
O4	0.086 (3)	0.083 (3)	0.0307 (18)	0.014 (2)	0.0110 (18)	0.0029 (18)
O5	0.0442 (16)	0.041 (2)	0.066 (2)	−0.0103 (14)	0.0161 (16)	−0.0047 (16)
N1	0.0356 (17)	0.036 (2)	0.0289 (18)	0.0011 (15)	0.0032 (15)	0.0021 (16)
N2	0.0412 (19)	0.033 (2)	0.036 (2)	−0.0059 (15)	0.0083 (16)	−0.0016 (16)
N3	0.0299 (15)	0.034 (2)	0.0332 (19)	−0.0005 (14)	0.0027 (15)	−0.0007 (16)
C1	0.055 (3)	0.040 (3)	0.038 (3)	0.001 (2)	0.008 (2)	0.001 (2)
C2	0.069 (3)	0.041 (3)	0.050 (3)	0.020 (2)	0.013 (3)	0.006 (2)
C3	0.056 (3)	0.064 (4)	0.060 (3)	0.028 (3)	0.024 (3)	0.025 (3)
C4	0.038 (2)	0.067 (4)	0.053 (3)	0.006 (2)	0.014 (2)	0.024 (3)
C5	0.035 (2)	0.045 (3)	0.026 (2)	0.0034 (18)	0.0040 (18)	0.0083 (19)
C6	0.040 (2)	0.043 (3)	0.025 (2)	−0.006 (2)	0.0081 (17)	0.003 (2)
C7	0.043 (2)	0.054 (3)	0.047 (3)	−0.014 (2)	0.007 (2)	0.001 (2)
C8	0.060 (3)	0.060 (4)	0.046 (3)	−0.032 (3)	0.011 (2)	−0.004 (3)
C9	0.075 (3)	0.036 (3)	0.056 (3)	−0.020 (2)	0.008 (3)	0.000 (2)
C10	0.051 (2)	0.039 (3)	0.053 (3)	−0.003 (2)	0.008 (2)	0.000 (2)
C11	0.037 (2)	0.045 (3)	0.030 (2)	−0.0020 (19)	0.0022 (18)	0.002 (2)
C12	0.051 (3)	0.057 (3)	0.048 (3)	0.014 (2)	−0.003 (2)	0.008 (3)
C13	0.046 (2)	0.053 (3)	0.066 (4)	0.018 (2)	0.011 (2)	0.006 (3)
C14	0.049 (2)	0.043 (3)	0.049 (3)	0.000 (2)	0.024 (2)	−0.006 (2)
C15	0.0354 (19)	0.036 (3)	0.029 (2)	−0.0064 (18)	0.0116 (17)	−0.0022 (19)
C16	0.051 (2)	0.055 (3)	0.036 (2)	−0.001 (2)	0.001 (2)	−0.001 (2)
C17	0.046 (2)	0.046 (3)	0.034 (2)	−0.005 (2)	0.008 (2)	0.005 (2)
C18	0.265 (17)	0.258 (19)	0.143 (13)	−0.065 (15)	0.017 (13)	−0.007 (12)
C19	0.201 (13)	0.137 (10)	0.160 (13)	0.007 (10)	0.031 (11)	−0.016 (10)
O6	0.092 (3)	0.137 (5)	0.116 (5)	−0.009 (3)	−0.002 (3)	−0.045 (4)

### Geometric parameters (Å, °)

**Table:**

V1—O5	1.586 (3)	C6—C7	1.391 (6)
V1—N3	2.020 (3)	C7—C8	1.375 (7)
V1—O1	2.021 (3)	C7—H7	0.9300
V1—O2	2.035 (3)	C8—C9	1.372 (8)
V1—N1	2.130 (3)	C8—H8	0.9300
V1—N2	2.304 (3)	C9—C10	1.386 (7)
O1—C16	1.297 (5)	C9—H9	0.9300
O2—C17	1.286 (6)	C10—H10	0.9300
O3—C16	1.225 (5)	C11—C12	1.376 (6)
O4—C17	1.224 (6)	C11—C16	1.495 (7)
N1—C5	1.353 (5)	C12—C13	1.384 (7)
N1—C1	1.354 (5)	C12—H12	0.9300
N2—C6	1.339 (5)	C13—C14	1.383 (7)
N2—C10	1.347 (6)	C13—H13	0.9300
N3—C15	1.336 (5)	C14—C15	1.380 (6)
N3—C11	1.339 (5)	C14—H14	0.9300
C1—C2	1.369 (7)	C15—C17	1.506 (6)
C1—H1	0.9300	C18—C19	1.451 (19)
C2—C3	1.359 (7)	C18—H18D	0.9600
C2—H2	0.9300	C18—H18B	0.9600
C3—C4	1.377 (7)	C18—H18C	0.9600
C3—H3	0.9300	C19—O6	1.359 (15)
C4—C5	1.384 (6)	C19—H19C	0.9700
C4—H4	0.9300	C19—H19B	0.9700
C5—C6	1.475 (6)	O6—H6	0.8200
			
O5—V1—N3	105.83 (15)	C8—C7—H7	120.6
O5—V1—O1	99.69 (15)	C6—C7—H7	120.6
N3—V1—O1	76.93 (13)	C9—C8—C7	119.9 (5)
O5—V1—O2	99.30 (16)	C9—C8—H8	120.0
N3—V1—O2	76.59 (12)	C7—C8—H8	120.0
O1—V1—O2	150.73 (13)	C8—C9—C10	118.2 (5)
O5—V1—N1	91.59 (15)	C8—C9—H9	120.9
N3—V1—N1	162.44 (14)	C10—C9—H9	120.9
O1—V1—N1	98.38 (13)	N2—C10—C9	122.8 (5)
O2—V1—N1	103.14 (12)	N2—C10—H10	118.6
O5—V1—N2	163.88 (15)	C9—C10—H10	118.6
N3—V1—N2	90.28 (13)	N3—C11—C12	120.0 (4)
O1—V1—N2	83.65 (13)	N3—C11—C16	111.0 (4)
O2—V1—N2	84.25 (13)	C12—C11—C16	129.0 (4)
N1—V1—N2	72.30 (13)	C11—C12—C13	118.6 (4)
C16—O1—V1	118.4 (3)	C11—C12—H12	120.7
C17—O2—V1	118.5 (3)	C13—C12—H12	120.7
C5—N1—C1	118.1 (4)	C14—C13—C12	120.6 (4)
C5—N1—V1	121.6 (3)	C14—C13—H13	119.7
C1—N1—V1	120.3 (3)	C12—C13—H13	119.7
C6—N2—C10	118.1 (4)	C15—C14—C13	118.3 (4)
C6—N2—V1	115.8 (3)	C15—C14—H14	120.8
C10—N2—V1	126.0 (3)	C13—C14—H14	120.8
C15—N3—C11	122.4 (4)	N3—C15—C14	120.1 (4)
C15—N3—V1	118.7 (3)	N3—C15—C17	111.4 (4)
C11—N3—V1	118.9 (3)	C14—C15—C17	128.5 (4)
N1—C1—C2	121.9 (5)	O3—C16—O1	123.8 (5)
N1—C1—H1	119.1	O3—C16—C11	121.6 (4)
C2—C1—H1	119.1	O1—C16—C11	114.5 (4)
C3—C2—C1	120.3 (5)	O4—C17—O2	126.0 (4)
C3—C2—H2	119.8	O4—C17—C15	120.3 (4)
C1—C2—H2	119.8	O2—C17—C15	113.6 (4)
C2—C3—C4	118.7 (4)	C19—C18—H18D	109.5
C2—C3—H3	120.7	C19—C18—H18B	109.5
C4—C3—H3	120.7	H18D—C18—H18B	109.5
C3—C4—C5	119.6 (5)	C19—C18—H18C	109.5
C3—C4—H4	120.2	H18D—C18—H18C	109.5
C5—C4—H4	120.2	H18B—C18—H18C	109.5
N1—C5—C4	121.4 (4)	O6—C19—C18	157.2 (13)
N1—C5—C6	115.0 (4)	O6—C19—H19C	97.0
C4—C5—C6	123.6 (4)	C18—C19—H19C	97.0
N2—C6—C7	122.1 (4)	O6—C19—H19B	97.0
N2—C6—C5	115.2 (4)	C18—C19—H19B	97.0
C7—C6—C5	122.7 (4)	H19C—C19—H19B	103.5
C8—C7—C6	118.8 (5)	C19—O6—H6	109.5
			
O5—V1—O1—C16	−98.6 (3)	V1—N1—C5—C4	−179.6 (3)
N3—V1—O1—C16	5.5 (3)	C1—N1—C5—C6	−178.4 (4)
O2—V1—O1—C16	31.2 (5)	V1—N1—C5—C6	1.3 (5)
N1—V1—O1—C16	168.3 (3)	C3—C4—C5—N1	−0.7 (7)
N2—V1—O1—C16	97.3 (3)	C3—C4—C5—C6	178.4 (4)
O5—V1—O2—C17	93.9 (3)	C10—N2—C6—C7	−0.6 (6)
N3—V1—O2—C17	−10.3 (3)	V1—N2—C6—C7	−178.7 (3)
O1—V1—O2—C17	−35.9 (5)	C10—N2—C6—C5	177.5 (4)
N1—V1—O2—C17	−172.2 (3)	V1—N2—C6—C5	−0.6 (5)
N2—V1—O2—C17	−101.9 (3)	N1—C5—C6—N2	−0.3 (5)
O5—V1—N1—C5	178.2 (3)	C4—C5—C6—N2	−179.5 (4)
N3—V1—N1—C5	−8.7 (6)	N1—C5—C6—C7	177.7 (4)
O1—V1—N1—C5	−81.7 (3)	C4—C5—C6—C7	−1.4 (7)
O2—V1—N1—C5	78.3 (3)	N2—C6—C7—C8	0.4 (7)
N2—V1—N1—C5	−1.2 (3)	C5—C6—C7—C8	−177.5 (4)
O5—V1—N1—C1	−2.1 (3)	C6—C7—C8—C9	−0.2 (8)
N3—V1—N1—C1	171.0 (4)	C7—C8—C9—C10	0.1 (8)
O1—V1—N1—C1	98.0 (3)	C6—N2—C10—C9	0.5 (7)
O2—V1—N1—C1	−102.0 (3)	V1—N2—C10—C9	178.4 (4)
N2—V1—N1—C1	178.5 (3)	C8—C9—C10—N2	−0.2 (8)
O5—V1—N2—C6	−1.2 (7)	C15—N3—C11—C12	1.5 (6)
N3—V1—N2—C6	178.7 (3)	V1—N3—C11—C12	179.3 (3)
O1—V1—N2—C6	101.8 (3)	C15—N3—C11—C16	−175.6 (4)
O2—V1—N2—C6	−104.9 (3)	V1—N3—C11—C16	2.2 (5)
N1—V1—N2—C6	0.9 (3)	N3—C11—C12—C13	−0.6 (7)
O5—V1—N2—C10	−179.1 (5)	C16—C11—C12—C13	175.9 (5)
N3—V1—N2—C10	0.7 (4)	C11—C12—C13—C14	−0.1 (8)
O1—V1—N2—C10	−76.1 (4)	C12—C13—C14—C15	−0.1 (7)
O2—V1—N2—C10	77.2 (4)	C11—N3—C15—C14	−1.7 (6)
N1—V1—N2—C10	−177.0 (4)	V1—N3—C15—C14	−179.5 (3)
O5—V1—N3—C15	−89.6 (3)	C11—N3—C15—C17	175.4 (4)
O1—V1—N3—C15	173.8 (3)	V1—N3—C15—C17	−2.4 (4)
O2—V1—N3—C15	6.4 (3)	C13—C14—C15—N3	1.0 (6)
N1—V1—N3—C15	97.6 (5)	C13—C14—C15—C17	−175.6 (4)
N2—V1—N3—C15	90.4 (3)	V1—O1—C16—O3	176.1 (4)
O5—V1—N3—C11	92.5 (3)	V1—O1—C16—C11	−6.0 (5)
O1—V1—N3—C11	−4.1 (3)	N3—C11—C16—O3	−179.7 (5)
O2—V1—N3—C11	−171.5 (3)	C12—C11—C16—O3	3.6 (8)
N1—V1—N3—C11	−80.3 (5)	N3—C11—C16—O1	2.4 (6)
N2—V1—N3—C11	−87.5 (3)	C12—C11—C16—O1	−174.4 (5)
C5—N1—C1—C2	−0.1 (6)	V1—O2—C17—O4	−168.8 (4)
V1—N1—C1—C2	−179.8 (4)	V1—O2—C17—C15	11.9 (5)
N1—C1—C2—C3	−0.5 (8)	N3—C15—C17—O4	174.6 (4)
C1—C2—C3—C4	0.5 (8)	C14—C15—C17—O4	−8.6 (7)
C2—C3—C4—C5	0.1 (8)	N3—C15—C17—O2	−6.1 (5)
C1—N1—C5—C4	0.7 (6)	C14—C15—C17—O2	170.8 (4)

### Hydrogen-bond geometry (Å, °)

**Table:**

*D*—H···*A*	*D*—H	H···*A*	*D*···*A*	*D*—H···*A*
O6—H6···O4^i^	0.82	2.00	2.815 (6)	173
C1—H1···O6^ii^	0.93	2.50	3.216 (7)	134
C4—H4···O1^iii^	0.93	2.47	3.207 (6)	137
C9—H9···O6^iv^	0.93	2.50	3.220 (9)	135
C12—H12···O5^v^	0.93	2.46	3.374 (5)	166
C14—H14···O5^vi^	0.93	2.52	3.146 (6)	125

Symmetry codes: (i) *x*−1/2, −*y*+1/2, *z*+1/2; (ii) *x*+1/2, *y*+1/2, *z*; (iii) −*x*+1, *y*, −*z*+1/2; (iv) *x*+1/2, *y*−1/2, *z*; (v) −*x*+3/2, *y*−1/2, −*z*+1/2; (vi) −*x*+3/2, −*y*+1/2, −*z*.
